# At-Home Telehealth-Supported Subcutaneous Ketamine Therapy for Safety, Feasibility, and Clinical Outcomes in Adults With Moderate to Severe Depression, Anxiety, or Posttraumatic Stress Disorder in a Large, Heterogeneous Cohort in the United States: Retrospective Cohort Study

**DOI:** 10.2196/92647

**Published:** 2026-07-20

**Authors:** Acacia C Parks, Amanda L Woodward, Robert D Henry, Kristin A Arden, Leonardo Vando, Jack Swain, Bishal Lamichhane

**Affiliations:** 1 Liquid Amber Watertown, MA United States; 2 TSET Health Promotion Research Center University of Oklahoma Health Sciences Center Tulsa, OK United States; 3 Department of Biostatistics & Epidemiology University of Oklahoma Health Sciences Center Tulsa, OK United States; 4 Mindbloom Orlando, FL United States; 5 Department of Psychiatry and Behavioral Health Houston Methodist Academic Institute (HMAI) Houston Methodist Houston, TX United States

**Keywords:** digital health, generalized anxiety disorder, ketamine, major depressive disorder, mental health, psychedelic, posttraumatic stress disorder, PTSD, real-world, telemedicine

## Abstract

**Background:**

Depression, anxiety, and posttraumatic stress disorder (PTSD) are leading global causes of disability. Standard interventions have slow mechanisms of action, high attrition, and significant accessibility barriers. While intravenous and intranasal ketamine are rapid-acting alternatives, high cost and intensive logistical requirements limit adoption. Sublingual at-home ketamine addresses some gaps but is constrained by low bioavailability and variable absorption. Subcutaneous administration offers high bioavailability and precise dosing, potentially bridging the gap between in-clinic effectiveness and at-home accessibility.

**Objective:**

This study evaluated the safety, feasibility, and clinical outcomes of a telehealth, at-home subcutaneous ketamine protocol using a convenience sample of deidentified health records collected through Mindbloom’s telehealth platform across 38 states.

**Methods:**

This retrospective cohort study analyzed deidentified health records from 3870 patients with moderate to severe symptoms of depression (Patient Health Questionnaire-9 [PHQ-9] score≥10), anxiety (Generalized Anxiety Disorder-7 Scale [GAD-7] score≥10), or PTSD (PTSD Checklist for *Diagnostic and Statistical Manual of Mental Disorders* [Fifth Edition; PCL-5] ≥33). Participants completed a structured program involving clinical assessment, mandatory peer monitoring, remote physiological screening, mailed injection kits and blood pressure monitors, and clinician-guided subanesthetic dosing starting at 0.5 mg/kg. Primary outcomes were measured at baseline and after weeks 2, 4, and 6 using the PHQ-9, GAD-7, and PCL-5. Linear mixed effects models with cubic splines analyzed symptom trajectories and accounted for time-varying assessments. Statistical significance was defined as α=.05; effect sizes were reported. Sensitivity analyses used multiple imputation and last observation carried forward.

**Results:**

Patients (mean age 44.7, SD 10.8 years; 1592/3041, 52.4% female) demonstrated high adherence, with 0.5% (16/3041) switching from subcutaneous to sublingual administration. After 6 sessions (approximately 44 days), adjusted marginal means showed significant declines: PHQ-9 scores dropped from 14.64 (95% CI 13.99-15.29) to 6.30 (95% CI 5.90-6.70), GAD-7 from 13.06 (95% CI 12.45-13.67) to 6.09 (95% CI 5.72-6.47), and PCL-5 from 46.7 (95% CI 43.30-50.10) to 27.5 (95% CI 25.40-29.70) with large effect sizes (1.35-1.58). Minimal clinically important difference was achieved by 81.8% (603/737) patients with major depressive disorder, 80% (475/594) with generalized anxiety disorder, and 84.6% (203/240) with PTSD (*P*<.001 for all). Adverse events were low (2.8%-3.2%), with no serious complications related to subcutaneous administration.

**Conclusions:**

This study is the first large-scale evaluation of at-home subcutaneous ketamine. Results suggest that at-home subcutaneous ketamine is a safe, feasible intervention associated with high rates of symptom reduction in depression, anxiety, and PTSD. It differs from existing literature by using a high-bioavailability (93%) subcutaneous route in a remote setting, whereas patients typically receive infusions of this potency in-clinic. Patients achieved clinical outcomes comparable to or exceeding traditional and intranasal therapies, potentially closing the access gap for treatment-resistant populations and supporting the expansion of supervised telehealth models in mental health care.

## Introduction

Despite decades of research into the development of medications and evidence-based psychotherapies for major depressive disorder (MDD), generalized anxiety disorder, and posttraumatic stress disorder (PTSD), these conditions remain leading causes of disability globally [1-3]. Compounding this burden, therapeutic breakthroughs in evidence-based care have not translated into broad clinical access, leaving the majority of individuals with these diseases without evidence-based, or even minimal, interventions [4,5]; with only 28% of the US population having access to mental health professionals in the needed numbers and specialties to meet patient needs [6].

While the “pot” of mental health in the United States had already been close to boiling in 2019 [7], the COVID-19 pandemic was the tipping point that drove home gaps in access to mental health treatment and led to more widespread adoption of telemedicine [8-10]. Prior to 2019, general sentiment was that use of telemedicine would yield lower quality care—but in practice, the data suggest otherwise, with standard of care delivered remotely being comparable to that delivered in-person [11,12]. The pervasiveness and acceptability of telemedicine are important first steps in closing gaps between evidence-based care and the people who need it, but it is only one step—because even if everyone in need could access standard of care, the standard of care is imperfect and in dire need of substantial improvement.

The clinical management of MDD, generalized anxiety disorder, and PTSD is hindered by slow mechanisms of action and varying response rates across first-line interventions. Fewer than 50% of patients with depression respond to psychotherapy, with only approximately one-third reaching remission [13]; similarly, nearly 50% of anxiety patients receiving cognitive behavioral therapy fail to achieve remission [14]. PTSD outcomes also remain suboptimal, with high nonresponse and dropout rates to first-line treatments [15,16]. Pharmacological outcomes across clinical conditions reflect comparable constraints, characterized by a significant attrition of patients at every stage of the treatment pipeline. Barriers begin before the first dose; the prevalence of unfilled prescriptions for new antidepressant therapy is as high as 34% [17], and 50% of patients who do start will discontinue therapy prematurely [18]. Consequently, while literature suggests fewer than 50% of patients respond by week 5 [19], these figures represent only the self-selected subset of patients who remained adherent. This pattern of attrition and modest therapeutic effects is mirrored in anxiety medications, where treatments often fail to produce reliable response and remission across patient populations [20], while certain anxiety medications, namely benzodiazepines, carry the additional risk of dependence and abuse [21]. Finally, pharmacological treatments for PTSD are characterized by small effect sizes and a notable lack of evidence for most of the available medications [22].

Standard antidepressant therapies are further limited by persistent side effects, including sexual dysfunction, bone density loss, and insidious effects like hyponatremia and emotional blunting, which can severely impair quality of life and compliance [23]. Long-term use is also linked to iatrogenic comorbidities, including metabolic, skeletal, and paradoxical psychological symptoms, that can persist after discontinuation and increase vulnerability to future relapse [24].

For those who do not respond to initial treatments, alternatives include electroconvulsive therapy and transcranial magnetic stimulation, but there are numerous barriers to access, including restrictive insurance mandates, high out-of-pocket costs, and intensive logistical requirements, rendering them impractical for many patients [25-27]. Electroconvulsive therapy is also associated with deterioration in general cognitive function and learning ability [28]. Consequently, patients face a costly and demoralizing “roll of the dice” hoping for the right treatment [29]. In short, despite many innovations in the treatment of MDD, generalized anxiety disorder, and PTSD, there remains significant room for improvement.

Ketamine, a noncompetitive N-methyl-D-aspartate receptor antagonist, has emerged as a viable treatment option for depressive and anxious symptoms, including trauma-related symptoms [30-32]. Unlike traditional antidepressants, ketamine works by rapid synaptogenesis and glutamate modulation, both of which result in near-immediate relief [33]. When administered in-clinic, ketamine is often delivered intravenously, as an off-label use of the US Food and Drug Administration–approved anesthetic and analgesic, or intranasally, as Spravato (esketamine). While both intravenous and intranasal (esketamine) modalities are effective, they are limited by significant logistical and financial barriers that are prohibitive for many [34]. Structural obstacles, such as limited insurance reimbursement and skewed provider density in urban areas, further marginalize patients in rural and low-income communities [35].

Additionally, intravenous ketamine typically yields a response rate of 53.5% after 1 month [34], and esketamine (Spravato) induction response rates also vary across studies, with reports of 28.4% [36] and 40.2% [37]. These figures highlight a gap between the high resource requirements of clinic-bound protocols, the impact these requirements have on treatment adherence, and the varied clinical response achieved during the first month of treatment.

Advancements in telemedicine and delivery systems have significantly expanded the availability of off-label at-home administration. While antidepressants and psychotherapy report real-world response rates in the mid-40% range [13,19], previous evaluations of this at-home model have demonstrated that a more accessible and comfortable framework can be provided without compromising clinical efficiency. Specifically, a large-scale study of at-home sublingual ketamine treatment reported response rates of 56.4% for depression and 56.1% for anxiety following 4 treatments in a treatment course spanning 4 weeks [38], comparable to response rates reported in intravenous ketamine studies [34], and both higher and more consistent than those reported for Spravato above. These results are made more clinically relevant by the speed at which they occurred—by 4 weeks, or approximately half the time required for standard treatments to take effect [19,39,40]. Given the rapidly growing evidence base for ketamine in the treatment of PTSD, these at-home treatment administration findings represent a promising framework for trauma-related symptoms as well [41].

While previous work has demonstrated that sublingual ketamine is associated with clinically meaningful improvement to patients, oral routes are inherently limited by low and inconsistent bioavailability [42]. Mindbloom [43] is a US-based mental health provider specializing in ketamine therapy. Their sublingual protocol instructs patients to hold tablets in their mouth for 7 minutes and then spit out saliva to avoid unwanted effects of swallowing medication, such as nausea and prolonged sedation, achieving a bioavailability of approximately 10%. In Mindbloom’s internal pilot data [43], only 42% of patients using sublingual tablets agreed that session intensity was consistent, and 31% reported treatments that were “too mild” to achieve therapeutic dissociation. Patients using the sublingual administration have also reported discomfort holding tablets in the mouth, and while rare (0.05% of individuals reported serious adverse events [SAEs]), 25% of SAEs with sublingual administration were driven by accidentally swallowing tablets [43].

To address the concerns raised by sublingual administration, subcutaneous injection provides an alternative option. Offering up to 93% bioavailability [44], subcutaneous injection can approximate the precision of in-clinic intravenous therapy while still being possible to administer at home [44]. Though this study is not the first to test subcutaneous ketamine injections, with some initial promising findings already published [45,46], it does represent a test of subcutaneous ketamine in a relatively large real-world sample. Previous publications from this group report on findings from sublingual ketamine use; this study presents entirely new data on patients using the subcutaneous mode of administration. The 2 primary hypotheses of the study are that subcutaneous injection is safe (hypothesis 1) and is associated with changes in clinical symptoms (hypothesis 2) when used as a mode of administration for at-home ketamine treatment. The secondary hypothesis is that subcutaneous injection is feasible for at-home use, marked by adherence and retention level (hypothesis 3). Because the study goals involve evaluating the at-home subcutaneous protocol as it is implemented in real-world practice, the retrospective observational approach is an appropriate vehicle for testing these hypotheses.

## Methods

### Setting

Study data were collected during Mindbloom’s clinical operations, a US-based direct-to-consumer organization specializing in ketamine therapy. Treatment is offered through a telehealth platform across 38 states, connecting patients with autonomous, licensed psychiatric clinicians. The Mindbloom platform can be accessed via an internet search or referral from an external physician/provider, while access to treatment through the platform is gated by rigorous screening from psychiatric clinicians.

### Inclusion and Exclusion

Patients in the analysis were a subset of Mindbloom patients seeking treatment for elevated symptoms of depression, anxiety, or PTSD, defined as a Patient Health Questionnaire-9 (PHQ-9) or Generalized Anxiety Disorder-7 Scale (GAD-7) score of ≥10, or a PTSD Checklist for *Diagnostic and Statistical Manual of Mental Disorders, Fifth Edition* (PCL-5) score of ≥33. Inclusion and exclusion criteria for treatment in the Mindbloom program are listed in Textbox 1.

In addition to the criteria in Textbox 1, patients with any of the following were evaluated for eligibility on a case-by-case basis and required to provide confirmation of concurrent, external therapeutic support to be considered for treatment: active moderate to severe alcohol or other substance use disorder, active moderate to severe opiate use disorder, and a history of severe trauma. In certain circumstances, additional medical clearance from outside providers and/or coordination with external mental health patients was required to ensure a patient was fit to proceed with treatment.

If a patient met initial eligibility requirements, they were invited to provide additional intake information. Patients filled out the new patient onboarding form through a patient portal, accessed via the web- and app-based Mindbloom platform, which included exclusion criteria screening tools and helped to prepare a clinician for an initial consultation. Clinicians assessed patient appropriateness for treatment using intake information, self-report assessments, a synchronous video consultation, and review of state prescription drug monitoring programs.

**Clinical inclusion and exclusion criteria for the at-home subcutaneous ketamine protocol.**

**Inclusion criteria**

- Diagnosis of anxiety, depressive disorder, and/or posttraumatic stress disorder
- >18 years of age
- Access to a safe, private environment for treatments and a peer treatment monitor
- No contraindications for treatment

**Exclusion criteria**

- Ketamine use disorder: mild, moderate, or severe. Active or in remission. Known hypersensitivity to ketamine
- Active psychotic or manic symptoms
- History of a primary psychotic disorder (eg, schizophrenia and schizoaffective disorder)
- Active suicidal ideation with method, intent, or plan in the past 3 months
- Suicide attempt within the past year
- Uncontrolled hypertension
- Congestive heart failure or other impaired cardiac status
- Severe and poorly-controlled respiratory problems (eg, chronic obstructive pulmonary disease)
- Untreated hyperthyroidism (laboratory work reviewed before proceeding)
- Elevated intraocular pressure (eg, glaucoma)
- Pregnant, nursing, or currently trying to become pregnant
- Other severe systemic disease not listed (at the clinician’s discretion)

### Participant Characteristics

Patients were aged 44.7 (10.8) years on average, and 52.4% (1592/3041) of the sample was female (1438/3041, 47.3% male, 8/3041, 0.3% “Prefer not to answer,” and 3/3041, 0.1% “Other”). The sample was 43.5% (1323/3041) married/partnered. Additionally, 71.8% (2183/3041) reported having a prior diagnosis of a psychological disorder, and 91.6% (2786/3041) reported receiving some form of prior treatment for a mental illness. Due to the retrospective nature of the study, some demographic information, such as ethnicity, was not available, as these variables were not collected in the regular course of care.

### Study Design

This study was a retrospective cohort study based on routinely collected clinical data from patients receiving telehealth-supported at-home subcutaneous ketamine treatment through the Mindbloom platform.

#### Protocol Development and Pilot History

Implementation of the at-home subcutaneous treatment model was preceded by an 11-month pilot initiative. The pilot consisted of 3 phases, and a medical review board of independent psychiatrists was created to review results of each pilot stage (side effects, adverse events, feasibility of at-home administration, symptom assessment scores, and qualitative patient and clinician feedback) and determine whether to move forward with broader implementation. Phase 1 included established sublingual patients who had not yet achieved a symptom response (n=49). Phase 2 included established sublingual patients who experienced administration challenges with sublingual tablets (n=277). Phase 3 included a combination of new and established patients (n=675). This pilot initiative formalized a protocol for at-home subcutaneous ketamine administration, which is further described below. Data from this initiative are not included in the analysis.

#### Sampling Procedures

Between January 2024 and October 2025, a total of 3870 patients were approved for subcutaneous treatment based on the study’s inclusion and exclusion parameters. The start date corresponded to when Mindbloom began offering subcutaneous ketamine. The end date was selected to ensure participants had sufficient time to complete the 6-session course examined by this study. Overall, 303 patients did not move forward with session 1, and so 3567 patients completed at least 1 treatment. A further 526 patients were excluded for exceeding a 90-day interval between baseline and session 2, leaving 3041 patients who were included in the overall analysis. As symptom progression and outcomes were analyzed separately across self-report measures, patient data for each self-report measure were excluded from analysis if they did not reach the threshold for moderate symptoms (ie, removed from the analysis pool for the given measure if below 10 for PHQ-9, below 10 for GAD-7, and below 33 for PCL-5 at baseline). In addition, patient data were excluded from the analysis of each self-report measure if no follow-up was reported for the given measure (ie, only a single assessment was available for a given patient), or if more than 90 days had passed between baseline and session 2.

#### Sample Size, Power, and Precision

Because this was a retrospective analysis on existing data, no power analysis was performed.

### Ethical Considerations

Prior to treatment, patients and clinicians gave passive consent by agreeing to user terms and conditions that included the use of deidentified, aggregated data for research purposes. Data were deidentified prior to analysis to ensure patient anonymity in accordance with HIPAA (Health Insurance Portability and Accountability Act) standards, and no identifiable information or images of individual participants are included in this manuscript. No compensation was provided to patients for the use of their clinical data. The analysis in this paper constituted a retrospective analysis of Mindbloom’s routinely collected clinical data, and was deemed institutional review board (IRB) exempt by Biomedical Research Alliance of New York (BRANY) IRB (BRANY File #25-12-706-2414). Patients completed informed consent for treatment. However, as this was formally determined by the IRB to be exempt from IRB review, the study therefore did not require informed consent for research participation, in compliance with *45 CFR part 46 Category 4: Common Rule, Secondary research*, for which consent is not required.

### Clinicians and Guides

Clinicians had current prescription privileges in their state of licensure, completed a 30-hour training on Mindbloom's protocols, and passed an evaluation of their psychiatric evaluation skills with a standardized patient. Regular chart reviews were also conducted, as well as clinical quality metric monitoring to identify and provide coaching opportunities. Weekly ongoing training was provided for advanced skills.

Each patient was assigned a behavioral coach, known as a “guide,” to provide coaching, patient education, and logistical support to facilitate treatment adherence and serve as a communication bridge to the clinical team. Guides were required to have a coaching certification and/or to have provided one-on-one behavioral coaching for more than 1 year with at least 50 clients.

### Intervention Phases and Clinical Execution

#### Treatment Steps

**Figure 1:**
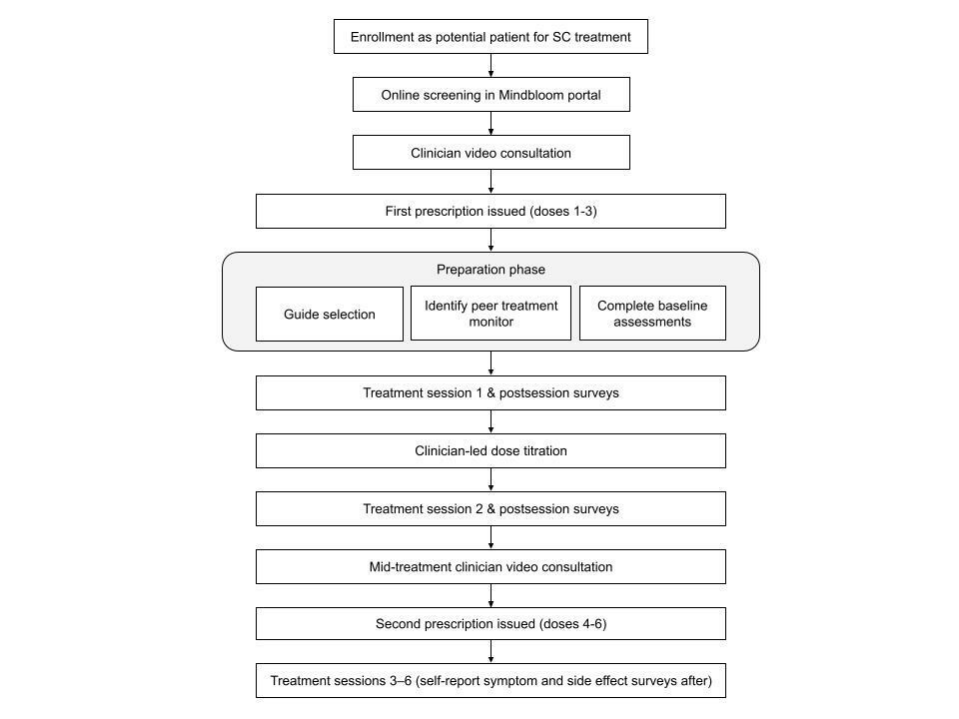
Operational workflow for the telehealth-supported subcutaneous ketamine treatment program.

Figure 1 outlines the steps in the treatment process from initial eligibility screening through the completion of 6 treatment sessions.

#### Preintervention Consultation and Preparation for Treatment

Patients who passed the initial eligibility screening participated in a comprehensive, synchronous video consultation for diagnostic verification and safety planning. This initial consultation involved collecting the History of Present Illness, completing the Mental Status Exam, a review of medical systems, a thorough review of family and medical and psychiatric history, a review of state-specific Prescription Drug Monitoring Program reports to assess controlled substance prescription history, and Surescripts reports to assess drug-drug interactions and complete medication reconciliation [47]. Eligibility was contingent upon the clinician’s verification of a safe home environment and the identification of a mandatory peer treatment monitor (PTM), a trusted adult who can be present for the duration of each session.

Following the initial consultation, patients were assigned a trained guide to facilitate preparation for treatment administration through a direct communication line and structured digital resources. Pretreatment requirements included the completion of baseline symptom assessments (1 or 2 of PHQ-9, GAD-7, and PCL-5).

After the clinician determined eligibility during the video consultation, patients were sent the first prescription batch of 3 doses, with a standard initial dose of 0.5 mg/kg (in line with standard intravenous dosing), and the ability for the clinician to titrate the dose as appropriate for subsequent doses. Patients received ondansetron (antinausea medication) 8 mg orally disintegrating tablet to be taken 1 hour prior to ketamine treatments unless any contraindications existed. After the first series of treatments, a video consultation occurred to establish the maintenance dosage for the final 3 treatments, and the next 3 doses were sent to the patient. Treatment renewals and dose adjustments occurred in concurrence with a video consultation with the clinician. While patients could continue treatment through subsequent renewals, the analysis focuses specifically on the clinical outcomes observed during the initial 6 sessions. The pharmacy provided a standardized injection kit including a sharps container, alcohol preparation pads, and insulin syringes. Patients also received a digital blood pressure (BP) monitor, with sessions only commencing if vitals were stable, defined as a BP below 150/100 mm Hg and a heart rate below 100 bpm, no changes to physical or mental health since the last appointment with a clinician, and adherence to fasting and substance-avoidance protocols.

#### Dosing and Titration

The subcutaneous dosing protocol was initiated at a subanesthetic dose of 0.5 mg/kg of body weight, grounded in standard intravenous benchmarks and assuming 93% bioavailability [44].

Doses were rounded to the nearest 10 mg increment to facilitate patient ease and accurate self-administration. Subsequent clinician-directed titration was determined by a triad of clinical factors: medication tolerability, symptom reduction, and subjective session intensity, with the protocol subject to refinement as patient outcomes and new literature were continuously evaluated.

Titration occurred in cautious increments of 10-20 mg. For the 6 sessions, the maximum dose was capped at 120 mg or 1.2 mg/kg, whichever was more conservative. For patients transitioning from prior intravenous or intramuscular settings, providers assessed historical session intensity, response, and side-effect profiles to tailor the initial at-home dose. Continued titration was permitted only if side effects remained nonexistent or transient and well-tolerated. See Table 1 for treatment response rules that governed dosing titration.

**Table 1:** Rules for clinician-directed dose titration based on session intensity scores (0-10) and self-reported mood improvement.

Session intensity score	Mood: no improvement	Mood: some improvement	Mood: significant improvement
0-2	+20 mg	+20 mg	No change
3-7	+10 mg	+10 mg	No change
8-10	No change	No change	No change

The financial requirement for each subcutaneous dose in this study ranged from US $165 to US $215, a significant reduction in cost when compared to traditional in-clinic intravenous infusions that typically cost between US $300 and US $690 per session [34].

#### Safety Protocols and Self-Administration Training

At-home self-administration of subcutaneous medications is a growing practice across a diverse range of conditions, including diabetes (insulin and glucagon-like peptide-1 agonists), autoimmune diseases (biologics), and hormonal deficiencies, with studies consistently demonstrating positive safety outcomes when accompanied by appropriate training and support [48,49]. Following World Health Organization best practices for safe injection and needle handling [50], patients in this study received education via verbal, written, and video formats that included information on-site selection, supply collection, site preparation, syringe preparation and dose confirmation, administration, and proper needle disposal.

Administration training for patients was delivered by video-based clinician instruction and included information on hand washing, aseptic technique (both with the vial and injection site), the prohibition of syringe reuse, verifying dose accuracy, proper storage of medication, and proper disposal of syringes to prevent needlesticks. Competency was verified through a mandatory “return demonstration,” where patients performed the injection process under guided supervision.

In addition to robust inclusion and exclusion criteria, clinical risk was mitigated through the mandatory presence of a PTM and a 15-hour daily clinician-on-call line. Safety was further supported by presession vital sign screenings, prophylactic antinausea medication, and longitudinal surveys to monitor for emergent conditions such as dependency or worsened mood. To prevent misuse, clinicians performed state-specific Prescription Drug Monitoring Program reviews prior to every order and limited prescriptions to the minimum medication quantity required for the treatment phase.

#### Treatment Sessions

During session 1, a guide verified the presence of the mandatory PTM and performed a final physiological screening, requiring a BP below 150/100 mm Hg and a heart rate below 100 bpm. Following standardized video education and a verified return demonstration of the subcutaneous injection technique, the patient self-administered the dose under PTM supervision with clinician-on-call support available. Post session, patients completed a 30-minute recovery and journaling period before rejoining the synchronous video call with the guide.

A mandatory asynchronous clinician check-in occurred 24-72 hours following session 1 to evaluate tolerability and establish a titration plan. Subsequent treatment sessions used the same safety and preparation standards, supported by longitudinal outcome tracking using the relevant PHQ-9, GAD-7, and PCL-5 assessments at baseline and after sessions 2 and 4 to measure rapid and compounding therapeutic benefits. The integration program included 2 personalized video coaching sessions designed to align patient experiences with their therapeutic goals and identify any necessary midprogram protocol adjustments for the clinician.

Clinical oversight and safety monitoring were conducted through multiple synchronous and asynchronous channels for the duration of the program. Patients could engage in daily text-based communication with assigned guides, who conducted regular check-ins to assess treatment response and potential adverse events. Follow-up symptom measures were collected via biweekly longitudinal surveys to monitor progress and any emerging side effects. Clinicians evaluated treatment tolerability and side effects following sessions 1 and 2, while the patient portal administered standardized side-effect queries after sessions 2 and 4. Data from these portal responses were integrated into medical team check-ins alongside symptom measures to determine if clinical escalation was required. Patients used the provided digital BP monitor for mandatory presession BP checks, and were prompted to schedule a check-in with their clinician based on their responses to their self-report session logs. A clinician-on-call line also remained available for urgent support during active treatment hours.

### Measures and Covariates

#### Overview

Psychometric assessments were introduced to patients as an important aspect of their care to facilitate goal setting and track progress. Patients chose to complete either one or two of the following 3 questionnaires at baseline, as well as after sessions 2 and 4, 2 weeks and 2 weeks after baseline. This approach allowed patients to self-select the assessments that were most relevant to them.

#### Depressive Symptoms

The PHQ-9 was used to assess the severity of depressive symptomatology [51]. This validated instrument measures the frequency of *DSM-IV* (*Diagnostic and Statistical Manual of Mental Disorders* [Fourth Edition]) depressive symptoms over the preceding 2 weeks, with items rated on a 4-point scale ranging from 0 (“not at all”) to 3 (“nearly every day”). Cumulative scores range from 0 to 27, and following established diagnostic standards, scores ≥10 served as the threshold for clinical depression [52]. Internal consistency was strong to excellent across all time points (McDonald ω ranging from 0.866 to 0.903).

#### Generalized Anxiety Symptoms

Anxiety severity was quantified using the GAD-7 [53]. Similar to the PHQ-9, this measure uses a 4-point scale to evaluate symptom frequency. The scale provides a maximum total score of 21, where a score of 10 or higher serves as the established psychometric cut-off for moderate generalized anxiety [54]. Internal consistency was strong to excellent across all time points (ω ranging from 0.899 to 0.923).

#### PTSD Symptoms

To evaluate trauma-related distress, the 20-item PCL-5 was used [55]. This self-report instrument quantifies the severity of the 20 *DSM-5* (*Diagnostic and Statistical Manual of Mental Disorders* [Fifth Edition]) symptoms over the preceding month using a 5-point scale (0-4). The measure maps symptoms across 4 symptom domains: intrusion, avoidance, negative alterations in cognition and mood, and marked alterations in arousal and reactivity. Internal consistency was excellent across all time points (ω ranging from 0.928 to 0.961).

### Side Effect Monitoring

Side effects were assessed through a single-item self-report measure administered after session 2 and again after session 4: “Have you noticed any issues with your physical or mental health since beginning treatment?” Response options included chest pain, lower abdominal pain, increased BP, shortness of breath, cravings for ketamine, memory loss, pain urinating, suicidal thoughts, none of the above, and a field to specify anything else. Any adverse events reported to the clinician or guide outside of this measure were recorded in the electronic health record.

To account for missing data patterns and isolate the specific relationship between the subcutaneous treatment protocol and symptom reduction, several level-2 person-level constant variables were operationalized as statistical covariates in the primary analyses. These included demographic characteristics (baseline age and biological sex), clinical history (presence of a prior psychiatric diagnosis), and treatment parameters (final titrated medication dosage). Including these covariates ensured that the longitudinal symptom trajectories were adjusted for baseline imbalances and potential attrition biases.

### Statistical Analyses

First, we calculated descriptive statistics (eg, mean, SD, median, and range) at baseline, assessment wave 1 (after 2 sessions), assessment wave 2 (after 4 sessions), and assessment wave 3 (after 6 sessions) for the primary outcomes of interest (PHQ-9, GAD-7, and PCL-5) as well as number of side effects at each assessment wave. No transformations or filtering of data points beyond what was stated in the participant flow diagram/inclusion criteria were performed. Second, we computed the percent change in each of the primary outcomes, relative to baseline, and ran paired *t* tests to compare each percent difference to baseline. Finally, we built random-intercept mixed models to control for the nonlinear effect of time (days) from baseline. Models included a cubic spline term (*df*=3) for time and level-1 (time-varying) terms for assessment wave, as well as level-2 (person-level constant) terms for age (grand-mean centered), sex, final dosage (grand-mean centered), and history of a psychiatric diagnosis. These covariates were included to both account for missingness and to rule out between-person demographic and dosage effects. Spline models significantly improved fit compared to linear-time models (*χ*^2^_2_>10.22; *P*<.007). By including the assessment wave in addition to the spline term for days, the model thus estimates a nonlinear change in outcome (days) as well as a linear change (assessment wave). Models were estimated for the 3 primary outcome variables: PHQ-9, GAD-7, and PCL-5. From these models, we estimated marginal means for each primary outcome at the median number of days for each assessment wave. This approach helps to account for the high variability in the number of days between assessment waves.

## Results

### Participant Flow

**Figure 2:**
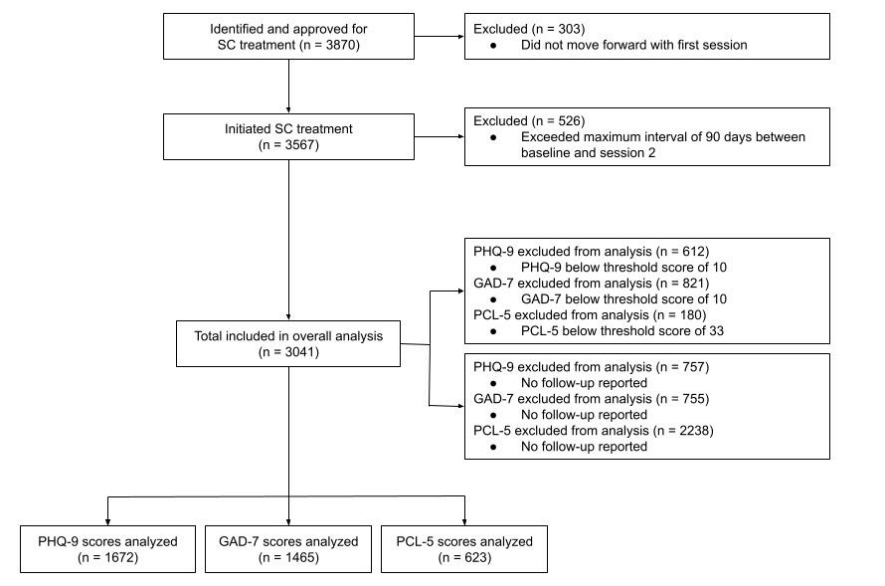
Participant flow diagram for inclusion in the analysis. GAD-7: Generalized Anxiety Disorder-7 Scale; PCL-5: Posttraumatic Stress Disorder Checklist for Diagnostic and Statistical Manual of Mental Disorders (Fifth Edition); PHQ-9: Patient Health Questionnaire-9; SC: subcutaneous.

A final sample of 3041 patients taking subcutaneous ketamine injections was included in this analysis (see Figure 2 for the participant flow diagram).

### Descriptive Statistics

We calculated the improvement in raw scores and percent change from baseline (Table 2). In this table, we calculated the number of people who experienced a minimal clinically important difference (MCID) in response to treatment in PHQ-9, GAD-7, and PCL-5. MCIDs were defined as improvements of 4 for PHQ-9 and GAD-7, and 5 or more points for the PCL-5.

**Figure 3:**
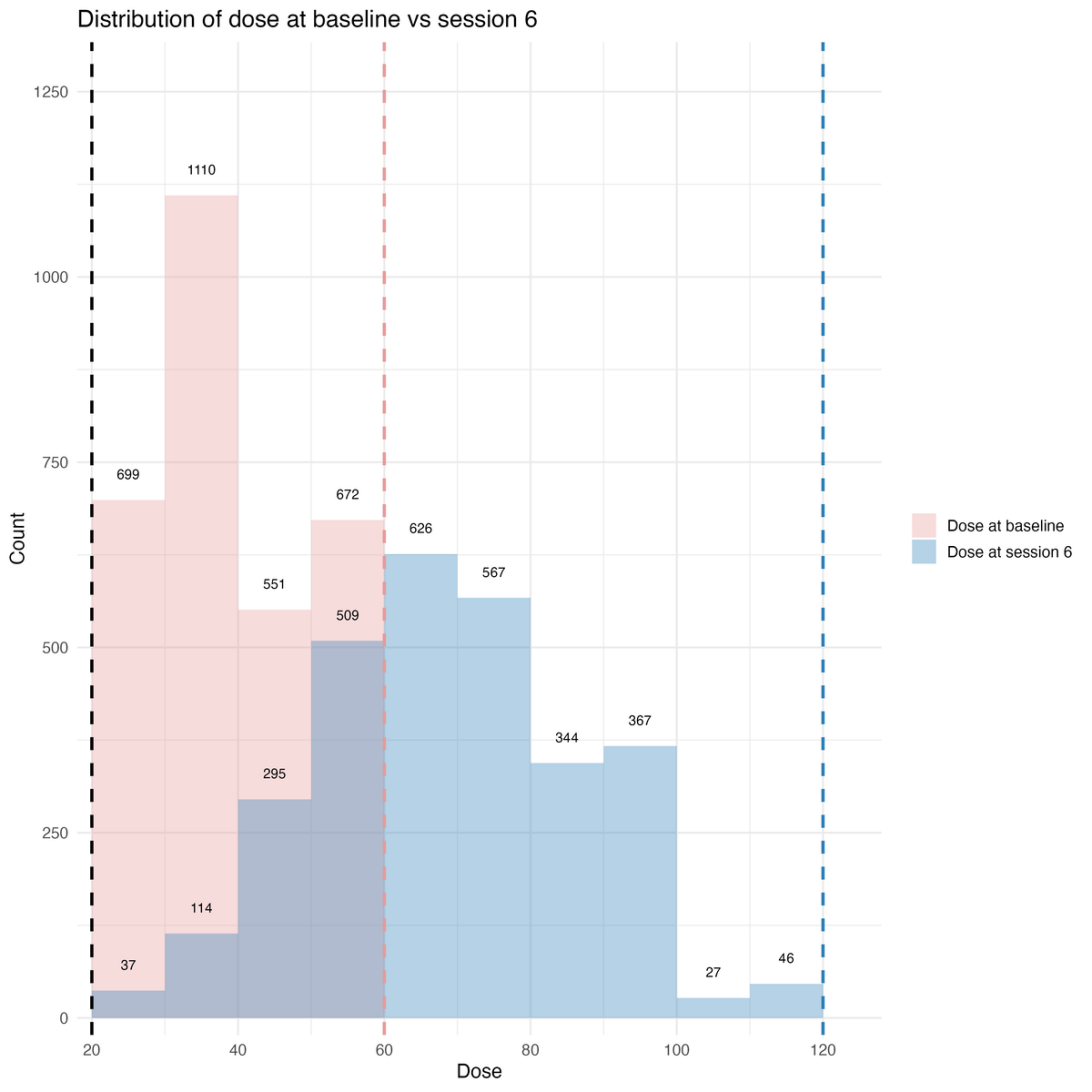
Comparison of subcutaneous ketamine dosage distributions at baseline and session 6. The black dashed line indicates the minimum dose administered at both time points (20 mg). Colored dashed lines represent the maximum doses for the respective baseline (red, 60 mg) and session 6 (blue, 120 mg) histograms.

Figure 3 shows the distribution of dose amount at baseline compared with session 6. Patients began the study at a median dose of 40.0 (IQR 10) mg and ended at a median dose of 70.0 (IQR 30) mg. This IQR calculation excludes the 0.4% of patients who transitioned from subcutaneous to sublingual administration prior to study completion.

**Table 2:** Raw descriptive statistics and percent change from baseline.

			Baseline		Assessment wave 1 (after 2 treatment sessions)		Assessment wave 2 (after 4 treatment sessions)		Assessment wave 3 (after 6 treatment sessions)
**PHQ-9^a^**									
	n	1672		1488		1174		737	
	Mean (SD)	15.8 (4.02)		9.57 (5.27)		8.48 (4.97)		7.74 (4.87)	
	Median (minimum-maximum)	15 (10-27)		9 (0-27)		8 (0-26)		7 (0-26)	
	% change from baseline	N/A^b^		–39.3		–46.1		–50.2	
	*P* value	N/A		<.001		<.001		<.001	
	MCID^c^, n (relative %)	N/A		1044 (70.2)		925 (78.8)		603 (81.8)	
	Response^d^, n (relative %)	N/A		623 (41.9)		606 (51.6)		417 (56.6)	
	Remission^e^, n (relative %)	N/A		239 (16.1)		265 (22.6)		204 (27.7)	
**GAD-7^e^**									
	n	1465		1274		1002		594	
	Mean (SD)	14.9 (3.35)		8.93 (4.97)		7.69 (4.60)		7.10 (4.55)	
	Median (minimum-maximum)	15 (10-21)		8 (0-21)		7 (0-21)		6 (0-21)	
	% change from baseline	N/A		–39.5		–47.7		–51.1	
	*P* value	N/A		<.001		<.001		<.001	
	MCID^c^, n (relative %)	N/A		871 (68.4)		784 (78.2)		475 (80)	
	Response^d^, n (relative %)	N/A		543 (42.6)		566 (54.5)		360 (60.6)	
	Remission^e^, n (relative %)	N/A		229 (18)		249 (24.9)		176 (29.6)	
**PCL-5^f^**									
	n	623		518		407		240	
	Mean (SD)	51.4 (11.2)		37.8 (16.0)		33.2 (16.4)		31.0 (16.6)	
	Median (minimum-maximum)	50 (33-80)		37 (0-80)		33 (0-77)		29 (0-80)	
	% change from baseline	N/A		–26.1		–35.4		–39.8	
	*P* value	N/A		<.001		<.001		<.001	
	MCID^c^, n (relative %)	N/A		373 (72)		329 (80.8)		203 (84.6)	
	Response^d^, n (relative %)	N/A		292 (56.4)		278 (68.3)		184 (76.7)	
	Remission^e^, n (relative %)	N/A		199 (38.4)		203 (49.9)		136 (56.7)	
**Days from baseline**									
	n	3041		3041		2387		1452	
	Mean (SD)	N/A		16.9 (12.3)		35.3 (20.3)		51.1 (29.4)	
	Median (minimum-maximum)	N/A		14 (4-88)		31 (4-299)		44 (16-341)	
	% change from baseline	—^g^		—		—		—	

^a^PHQ-9: Patient Health Questionnaire-9.

^b^N/A: not applicable.

^c^MCID: minimal clinically important difference; defined as an improvement of 4+ points for PHQ-9/GAD-7 scores, and 5+ points for PCL-5 scores.

^d^Response is defined as 50% or greater improvement in symptoms for PHQ-9/GAD-7, and a 10+ points improvement for PCL-5.

^e^Remission was defined as a final symptom score below 5 for PHQ-9/GAD-7; for PCL-5, Remission was defined as a 10+ pt improvement combined with a final score below 33.

^e^GAD-7: Generalized Anxiety Disorder-7 Scale.

^f^PCL-5: *PTSD Checklist for Diagnostic and Statistical Manual of Mental Disorders* (Fifth Edition).

^g^Not available.

### Side Effects and Safety

A total of 93 (3.1%) people reported side effects after session 2 (wave 1 of surveys), 76 (3.2%) people reported side effects after session 4 (wave 2 of surveys), and 41 (2.8%) people reported side effects after session 6 (wave 3 of surveys). The most common side effects at each assessment wave were as follows: lower abdominal pain (19/93 after session 2), lower abdominal pain (12/76 after session 4), and memory loss (14/41 after session 6). There was no association between dosage and risk for experiencing a side effect at either of these assessment waves (*P*>.17). Additionally, only 19 (0.6%) patients switched from subcutaneous to sublingual administration of ketamine by the conclusion of the study, and only 6 (0.2%) reported side effects from the injections themselves (eg, pain, swelling, and discomfort).

A total of 3 SAEs were reported during the 22-month reporting period of the study, for an SAE rate of 0.08% (3/3943). One patient experienced a psychotic episode following their first treatment and was determined to be likely related to ketamine treatment. Two patients died by suicide during treatment—one following their first treatment, and one following their second treatment—but the attributability of the deaths to treatment could not be determined.

### Mixed Models for Symptom Progression

**Figure 4:**
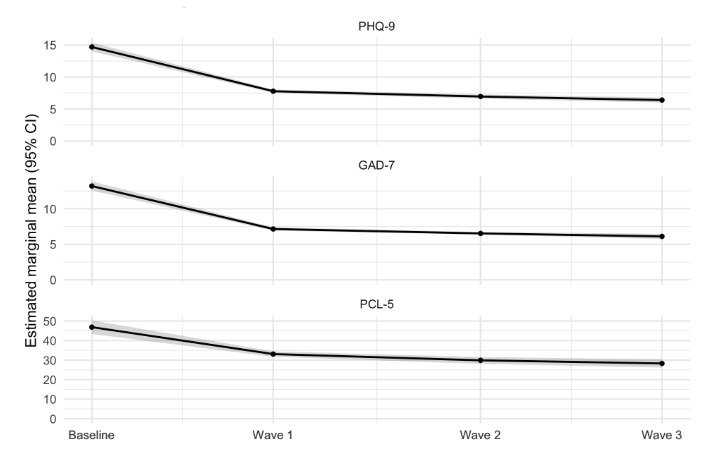
Estimated marginal means (95% CI) for Generalized Anxiety Disorder-7 Scale (GAD-7), Posttraumatic Stress Disorder Checklist for Diagnostic and Statistical Manual of Mental Disorders (Fifth Edition; PCL-5), and Patient Health Questionnaire-9 (PHQ-9) symptom trajectories over time (wave 1=after 2 sessions; wave 2=after 4 sessions; wave 3=after 6 sessions).

Separate models were run for PHQ-9, GAD-7, and PCL-5 scores, and each included random intercepts. All models controlled for nonlinear (spline) effects of time (days), since there were expected to be nonlinear and meaningful effects of time between each session. We also included the effect of assessment waves 1, 2, and 3, which occurred after sessions 2, 4, and 6, respectively. The full mixed model results are presented in Tables S1-S3 in Multimedia Appendix 1. Models showed that the assessment wave was significantly associated with consistent improvements in PHQ-9, GAD-7, and PCL-5 scores (see Table 3 for marginal means and Figure 4 for estimated marginal means over time). We also calculated cumulative within-person standardized differences in each outcome as the mean paired difference divided by the SD of each paired difference (ie, Cohen *d*–like effect sizes for mixed models, relative to baseline). Results indicate that effects were large and increased modestly across each assessment wave. CIs indicate relatively little variability in treatment outcomes, suggesting that the means are representative of what the majority of patients experienced.

**Table 3:** Adjusted marginal means (95% CIs) and effect sizes at each time point.

		Baseline (0 days)	Assessment wave 1 (14 days)	Assessment wave 2 (31 days)	Assessment wave 3 (44 days)
**PHQ-9^a^**					
	Mean (95% CI)^b^	14.64 (13.99-15.29)	7.74 (7.49-7.99)	6.85 (6.52-7.19)	6.30 (5.90-6.70)
	*P* value	N/A^c^	<.001	<.001	<.001
	*d_z_* ^d^	N/A	1.25	1.46	1.55
**GAD-7^e^**					
	Mean (95% CI)^b^	13.06 (12.45-13.67)	7.11 (6.88-7.34)	6.53 (6.22-6.84)	6.09 (5.72-6.47)
	*P* value	N/A	<.001	<.001	.005
	*d_z_* ^d^	N/A	1.21	1.52	1.58
**PCL-5^f^**					
	Mean (95% CI)^b^	46.7 (43.3-50.1)	32.8 (31.3-34.2)	29.4 (27.6-31.2)	27.5 (25.4-29.7)
	*P* value	N/A	<.001	<.001	.043
	*d_z_* ^d^	N/A	0.94	1.2	1.35

^a^PHQ-9: Patient Health Questionnaire-9.

^b^Means and 95% CIs are calculated at the median number of days for each respective time point based on the multilevel spline model. Estimated effects are at the population-level for simplicity.

^c^N/A: not applicable.

^d^Within-person standardized mean difference (*d_z_*) values indicate the standardized and adjusted effect of each assessment wave relative to baseline.

^e^GAD-7: Generalized Anxiety Disorder-7 Scale.

^f^PCL-5: *Posttraumatic Stress Disorder Checklist for Diagnostic and Statistical Manual of Mental Disorders* (Fifth Edition).

With respect to covariate effects, history of a psychological disorder was associated with higher overall scores on all 3 measures (*P*<.002) and final dose was associated weakly with overall higher PHQ-9 (*b*=.003; *P*=.02) and GAD-7 scores (*b*=.003; *P*=.01). Additionally, age predicted lower overall GAD-7 scores (*b=*-0.03; *P*<.001) and females reported higher PHQ-9 scores (*b*=.48; *P*=.007).

### Handling of Missing Data

With respect to attrition (ie, declining to complete surveys at future assessment waves), we found that female patients were slightly more likely to drop out (nonstatistically significant after 6 treatment sessions; 290/540, 53.8% of assessment wave 3 missing participants were female; *P*=.20). Reporting a psychological disorder at baseline was also associated with a slightly higher chance of attrition. Specifically, 73.9% (399/540) of those missing self-reports after 6 treatment sessions reported a disorder compared to 71.3% (1036/1452) of patients with a complete dataset across all assessment waves; *P*=.02. Given these associations with missingness, we can cautiously infer missingness at random; we included biological sex and previously reported psychological disorder diagnosis in our mixed models as covariates to account for this, as well as final dose and age, to rule out between-person effects of age and dosage. This statistical approach is consistent with established methodologies [31,38], and our specific analysis was modeled after the framework established by Hull et al [31]. Little missing completely at random (MCAR) test was run to assess if data were missing completely at random. The test revealed that outcome data were likely not MCAR (*χ*^2^_24_=389; *P*<.001). From these analyses, we cautiously conclude that the data are missing at random, but not MCAR.

To address concerns about bias introduced by missing data, we completed a more rigorous multilevel multiple imputation on our full dataset. The imputed data yielded quite similar results as the original results (see Table S4 in Multimedia Appendix 1 for the sensitivity analysis of descriptive statistics and clinical outcomes using multiple imputation). For further assurance, we conducted a “worst-case imputation” by carrying the last observation forward to all missing cases within a person (ie, last observation carried forward). This approach—which assumes that all patients who dropped out did so because they failed to improve and would not have improved had they stayed—yielded results that were similar to the primary model estimates, though, as expected, the last observation carried forward results showed that participants responded less over time.

Given that the results did not change substantially in either of these increasingly conservative sensitivity analyses, the original analysis was retained to allow for the ease of interpretation that comes with reporting raw means.

## Discussion

### Principal Findings

Consistent with our primary aims, this large-scale retrospective study supports our core hypotheses regarding the implementation of at-home subcutaneous ketamine therapy and the potential clinical impact of a telehealth-mediated subcutaneous ketamine protocol in a large, real-world cohort. First, the protocol demonstrated a high safety ceiling characterized by a low incidence of adverse effects and an exceptionally low rate of serious complications. Second, the mode of administration was associated with rapid, statistically significant, and clinically meaningful symptom reductions across all 3 evaluated psychological domains: depression, anxiety, and trauma-related distress. Finally, the secondary feasibility hypothesis was validated by robust treatment completion rates, high program adherence, and minimal patient-initiated transitions to alternative administration routes. Taken together, these findings indicate that a supervised, remote subcutaneous ketamine treatment can deliver rapid clinical improvements comparable to traditional clinic-bound therapies while maintaining an acceptable safety profile.

The safety and tolerability profile observed in this study represents a meaningful contribution to the clinical management of treatment-resistant mood conditions. The low rate for side effects stands in contrast to standard-of-care pharmacological alternatives [56]; although an exact side-by-side comparison cannot be made, it is difficult to ignore that the reported value for the former is orders of magnitude lower than the latter. The high degree of tolerability likely reduced barriers to follow-through, as only a minority of patients choose to transition to the sublingual option. The low adverse event rates indicate that remote risk can be effectively mitigated when patient self-administration training is standardized through a combination of well-established best practices [48-50], including comprehensive video instruction, a verified live demonstration, a mandatory adult monitor, and active clinician-directed titration rules.

Crucially, subcutaneous administration was shown to be associated with rapid and clinically significant symptom reduction. The majority of both depression and anxiety patients achieved an MCID by the first assessment wave, and treatment response by the second assessment wave, suggesting that the observed benefits of subcutaneous ketamine were not only statistically significant but large enough to be meaningfully felt by participants [51,52,55]. The symptom improvement observed in the PTSD cohort is particularly noteworthy, as this population often faces some of the highest rates of treatment resistance and dropout in traditional psychotherapy and pharmacological models [15]. Further, symptom reduction effects were evident early in the treatment course, highlighting the rapid onset of action observed in the sample. In a clinical landscape where standard-of-care antidepressants often require 1-2 months to show effectiveness [19], an accelerated window for meaningful improvement could be impactful for patients in acute distress.

Finally, the results indicate that self-administered subcutaneous injections are a feasible modality for remote, at-home clinical settings. While sublingual tablets are an appealing option for some patients, prior literature notes high variability in ketamine effects due to inconsistent sublingual absorption and discomfort with sublingual administration [43]. Our findings demonstrate that patients can use higher-bioavailability subcutaneous administration [44] at home with a low incidence of administration challenges or side effects and high rates of adherence. Given that patients who used subcutaneous ketamine at home largely did so without issue, these data align with the broader literature [45,46] to suggest that subcutaneous has the potential to provide a viable option alongside other modes of administration. The existence of multiple effective at-home approaches further validates the overall feasibility of the at-home telehealth treatment model [12].

The analysis represents what is, to our knowledge, the first published study containing data on at-home subcutaneous ketamine use for depression, anxiety, and PTSD. Using a massive, nationwide cohort that spans the majority of US states and includes robust samples of multiple psychiatric disorders, these data, while limited by their observational nature, provide valuable insight into the safety, feasibility, and observed clinical outcomes associated with subcutaneous ketamine delivered at home.

### Limitations

This study is a retrospective, observational cohort study and lacks a comparison arm with which to make any claims of superiority over other treatments. There are inherent limitations to using real-world data, where patients navigate care freely, and provide data only while an active patients. We were unable to analyze the impact of ethnicity, as collecting this variable was not a part of the clinic’s standard workflow; without these data points, we cannot evaluate potential group-level differences in the speed or size of treatment response, limiting generalizability. Lack of data on patients who leave treatment means higher attrition than a controlled trial, limiting our ability to assess potential biases introduced by attrition that may or may not be random. Another important limitation relates to safety; while SAEs were infrequent and could not be conclusively tied to (or ruled out from being related to) ketamine treatment, the occurrence of 2 suicides early in treatment underscores the importance of rigorous monitoring and safety planning, particularly during the induction phase.

One important interpretation note on our findings concerns the variability in assessment wave timing. Because participants reached each assessment wave at variable times (ie, differing numbers of days), models included a nonlinear spline for days from baseline. The model-based expected symptom scores (Figure 4) reflect changes associated with progression through sessions, while simultaneously accounting for heterogeneous time between sessions via the spline term. This approach allowed us to detect trends in the marginal means across waves and disseminate highly interpretable findings. However, it does not allow us to pinpoint the exact impact of treatment timing itself on psychometric outcomes, highlighting the need for future research with stricter time-anchored treatment protocols.

While there are limitations to the approach used in this paper, there are also great benefits to real-world observational designs; they provide data representative of real-world practice, and focus on the types of patients who would naturally gravitate toward at-home ketamine, rather than a sample that may never have naturally chosen this treatment modality. Thus, this design is sufficient to establish a baseline of safety and feasibility, as well as initial evidence of clinical use and observed outcomes that can be built on in future research.

Additional studies may disentangle the effect of support from guides and clinicians from the impact of ketamine itself; the relative roles of therapeutic “integration” and the physiological impact of ketamine on psychiatric symptoms; and the extent to which the results observed in the users accessing Mindbloom may be biased due to the self-selected nature of the sample.

### Conclusions

In summary, this paper demonstrates that telehealth protocols can safely implement a high-bioavailability injection model for home ketamine administration, at scale, without causing serious clinical complications. The broader implications of these findings challenge the assumption that exists, among some practitioners, that ketamine can only be used when closely supervised in a clinic setting [57]. Specifically, our findings suggest that remote guardrails, such as remote clinician check-ins, video-verified self-injection competency, and the provision of BP monitoring equipment at home, can effectively decentralize ketamine care without compromising patient safety. When administered at subanesthetic dosages, with strategies in place to provide clear instruction in a remote context, these data are suggestive of clinical benefit and acceptable safety outcomes, even in the absence of the costly and complex risk evaluation and mitigation strategy requirements that govern use of Spravato [58].

## Appendix

Multimedia Appendix 1 Additional tables.

## Abbreviations

BP: blood pressure
BRANY: Biomedical Research Alliance of New York
DSM-5: Diagnostic and Statistical Manual of Mental Disorders (Fifth Edition)
DSM-IV: Diagnostic and Statistical Manual of Mental Disorders (Fourth Edition)
GAD-7: Generalized Anxiety Disorder-7 Scale
HIPAA: Health Insurance Portability and Accountability Act
IRB: institutional review board
MCAR: missing completely at random
MCID: minimal clinically important difference
MDD: major depressive disorder
PCL-5: Posttraumatic Stress Disorder Checklist for Diagnostic and Statistical Manual of Mental Disorders (Fifth Edition)
PHQ-9: Patient Health Questionnaire-9
PTM: peer treatment monitor
PTSD: posttraumatic stress disorder
SAE: serious adverse event

## Data Availability

Protected patient data can be made available upon reasonable request to the corresponding author, with a statistical analysis plan, and a fully executed data use agreement.
