# Supplementary material for: At-Home Telehealth-Supported Subcutaneous Ketamine Therapy for Safety, Feasibility, and Clinical Outcomes in Adults With Moderate to Severe Depression, Anxiety, or Posttraumatic Stress Disorder in a Large, Heterogeneous Cohort in the United States: Retrospective Cohort Study
Source: J Med Internet Res. 2026 Jul 20;28:e92647. doi: 10.2196/92647 (PMC13434254; doi:10.2196/92647)
Supplement: Supplementary file 1 [file jmir_v28i1e92647_app1.docx]

**Table S1:** *PHQ-9: Mixed Model with Spline Term for Time Since Baseline.*

| **Parameter** | ***b* (SE)** | ***b* 95% CI** | | ***t*** | ***P*** |
| --- | --- | --- | --- | --- | --- |
| Intercept | 13.90 (0.22) | [13.47, 14.33] | | 62.86^***^ | < .001 |
|  | | | | |  |
| *Level-1 predictors (time-varying by person)* | | | | |  |
| Days Since Baseline (spline, knot 1)^a^ | -0.53 (0.71) | [-1.92, 0.85] | | -0.76 | .437 |
| Days Since Baseline (spline, knot 2)^a^ | -7.97 (0.82) | [-9.59, -6.36] | | -9.68^***^ | < .001 |
| Days Since Baseline (spline, knot 3)^a^ | 1.04 (1.45) | [-1.80, 3.88] | | 0.72 | .483 |
| Wave (post-session 2, 4, and 6) | -0.77 (0.10) | [-0.97, -0.58] | | -7.68^***^ | < .001 |
|  |  |  | |  |  |
| *Level-2 predictors (constant within a person)* | | | | |  |
| Age (grand-mean centered) | -0.002 (0.003) | [-0.009, 0.004] | | -0.73 | .001 |
| Final Dosage (grand-mean centered) | 0.03 (0.01) | [0.002, 0.056] | | 2.11^*^ | .037 |
| Previous Psychiatric Diagnosis (“Yes” = 1) | 1.58 (0.21) | [1.17, 1.99] | | 7.57^***^ | < .001 |
| Sex (“Female” = 1) | 0.48 (0.18) | [0.13, 0.83] | | 2.69^**^ | .006 |
| **Random Effects** | **Variance** | ***SD*** | |  |  |
| Intercept | 12.76 | 3.57 | |  |  |
| Residual | 10.02 | 3.17 | |  |  |
| **Model Fit** |  |  | |  |  |
| -2*Log Likelihood | -17749.2 | |  |  |  |
| Deviance | 35498.3 | |  |  |  |
| AIC / BIC | 35520.3 / 35594.5 | |  |  |  |
| Adjusted ICC | 0.56 | |  |  |  |

*Note*. Level-2 predictors are either dummy-coded or grand-mean centered.

^*^*p* < .05, ^**^*p* < .01, ^***^*p* < .001. ^a^Spline coefficients are not directly interpretable.

**Table S2:** *GAD-7: Mixed Model with Spline Term for Time Since Baseline*

| **Parameter** |  | ***b* (SE)** | ***b* 95% CI** | | ***t*** | ***P*** |
| --- | --- | --- | --- | --- | --- | --- |
| Intercept |  | 13.28 (0.20) | [12.89, 13.67] | | 66.96^***^ | < .001 |
|  | | | | |  |  |
| *Level-1 predictors (time-varying by person)* | | | | |  |  |
| Days Since Baseline (spline, knot 1)^a^ |  | -0.94 (0.67) | [-2.26, 0.38] | | -1.40 | .163 |
| Days Since Baseline (spline, knot 2)^a^ |  | -9.22 (0.80) | [-10.80, -7.64] | | -11.47^***^ | < .001 |
| Days Since Baseline (spline, knot 3)^a^ |  | -0.49 (1.41) | [-3.25, 2.28] | | -0.35 | .729 |
| Wave (post-session 2, 4, and 6) |  | -0.57 (0.10) | [-0.76, -0.38] | | -5.89^***^ | < .001 |
|  | | | | | |  |
| *Level-2 predictors (constant within a person)* | | | | | |  |
| Age (grand-mean centered) |  | -0.03 (0.01) | [-0.04, -0.01] | | -3.60^***^ | < .001 |
| Final Dosage (grand-mean centered) |  | 0.03 (0.01) | [0.005, 0.06] | | 2.31^*^ | .021 |
| Previous Psychiatric Diagnosis (“Yes” = 1) |  | 1.29 (0.18) | [0.93, 0.06] | | 7.09^***^ | < .001 |
| Sex (“Female” = 1) |  | -0.04 (0.15) | [-0.36, 0.28] | | -0.22 | .824 |
|  |  |  |  | |  |  |
| **Random Effects** |  | **Variance** | ***SD*** | |  |  |
| Intercept |  | 10.17 | 3.19 | |  |  |
| Residual |  | 9.17 | 3.03 | |  |  |
| **Model Fit** |  |  |  | |  |  |
| -2*Log Likelihood |  | -16743.9 | |  |  |  |
| Deviance |  | 33487.9 | |  |  |  |
| AIC / BIC |  | 33509.9 / 33583.6 | |  |  |  |
| Adjusted ICC |  | 0.53 | |  |  |  |

*Note*. Level-2 predictors are either dummy-coded or grand-mean centered.

^*^*p* < .05, ^**^*p* < .01, ^***^*p* < .001. ^a^Spline coefficients are not directly interpretable.

**Table S3:** *PCL-5: Mixed Model with Spline Term for Time Since Baseline*

| **Parameter** | ***b* (SE)** | ***b* 95% CI** | | ***t*** | ***P*** |
| --- | --- | --- | --- | --- | --- |
| Intercept | 47.86 (1.29) | [45.33, 50.38] | | 37.23^***^ | < .001 |
|  | | | |  |  |
| *Level-1 predictors (time-varying by person)* | | | |  |  |
| Days Since Baseline (spline, knot 1)^a^ | -10.51 (4.43) | [-19.21, -1.18] | | -2.37^*^ | .018 |
| Days Since Baseline (spline, knot 2)^a^ | -10.44 (8.70) | [-27.53, 6.62] | | -1.20 | .232 |
| Days Since Baseline (spline, knot 3)^a^ | 20.69 (18.22) | [-15.04, 56.41] | | 1.14 | .256 |
| Wave (post-session 2, 4, and 6) | -2.81 (0.53) | [-3.84, -1.78] | | -5.34^***^ | < .001 |
|  |  |  | |  |  |
| *Level-2 predictors (constant within a person)* | | | | |  |
| Age (grand-mean centered) | -0.01 (0.02) | [-0.04, 0.02] | | -0.41 | .306 |
| Final Dosage (grand-mean centered) | 0.06 (0.04) | [-0.02, 0.14] | | 1.44 | .153 |
| Previous Psychiatric Diagnosis (“Yes” = 1) | 3.67 (1.16) | [1.39, 5.95] | | 3.16^**^ | .002 |
| Sex (“Female” = 1) | 0.99 (1.05) | [-1.06, 3.05] | | 0.95 | .349 |
|  |  |  | |  |  |
| **Random Effects** | **Variance** | ***SD*** | |  |  |
| Intercept | 140.98 | 11.87 | |  |  |
| Residual | 90.34 | 9.50 | |  |  |
| **Model Fit** |  |  | |  |  |
| -2*Log Likelihood | -8577.9 | |  |  |  |
| Deviance | 17155.8 | |  |  |  |
| AIC / BIC | 17177.8 / 17240.3 | |  |  |  |
| Adjusted ICC | 0.61 | |  |  |  |

*Note*. Level-2 predictors are either dummy-coded or grand-mean centered.

^*^*p* < .05, ^**^*p* < .01, ^***^*p* < .001. ^a^Spline coefficients are not directly interpretable.

**Table S4:** *Sensitivity analysis of descriptive statistics and clinical outcomes using multiple imputation*

|  | Baseline | Assessment wave 1 (after 2 treatment sessions) | Assessment wave 2 (after 4 treatment sessions) | Assessment wave 3 (after 6 treatment sessions) |
| --- | --- | --- | --- | --- |
| **PHQ-9^d^** |  |  |  |  |
| *M (SD)* | 15.1 (4.56) | 8.48 (5.26) | 7.32 (4.89) | 5.42 (4.41) |
| *Mdn* | 15 | 7 | 6 | 4 |
| Min, Max | 10, 27 | 0, 27 | 0, 26 | 0, 26 |
| % change from baseline | N/A | -43.8% | -51.5% | -64.1% |
| *P* value | N/A | < .001 | < .001 | < .001 |
| *n (relative %) MCID^a^* | N/A | 1,044 (62.4%) | 1,164 (69.6%) | 1,176 (70.3%) |
| *n (relative %) response^b^* | N/A | 623 (37.3%) | 745 (44.6%) | 777 (46.5%) |
| *n*  *(relative %) remission^c^* | N/A | 239 (14.3%) | 315 (18.8%) | 349 (20.9%) |
| **GAD-7^e^** |  |  |  |  |
| *M (SD)* | 14.0 (4.12) | 7.7 (4.97) | 6.6 (4.58) | 4.79 (4.11) |
| *Mdn* | 14 | 7 | 6 | 4 |
| Min, Max | 10, 21 | 0, 21 | 0, 21 | 0, 21 |
| % change from baseline | N/A | -45.0% | -52.9% | -65.8% |
| *P* value | N/A | < .001 | < .001 | < .001 |
| *n (relative %) MCID^a^* | N/A | 871 (59.5%) | 973 (66.5%) | 983 (67.1%) |
| *n (relative %) response^b^* | N/A | 543 (37.1%) | 685 (46.8%) | 714 (48.7%) |
| *n (relative %) remission^c^* | N/A | 229 (15.6%) | 291 (19.9%) | 315 (21.5%) |
| **PCL-5^f^** |  |  |  |  |
| *M (SD)* | 49.8 (12.2) | 32.7 (16.5) | 28.4 (16.2) | 21.7 (15.5) |
| *Mdn* | 50 | 32 | 26 | 19 |
| Min, Max | 33, 80 | 0, 80 | 0, 78 | 0, 80 |
| % change from baseline | N/A | -34.3% | -41.0% | -56.4% |
| *P* value | N/A | < .001 | < .001 | < .001 |
| *n (relative %) MCID^a^* | N/A | 373 (59.9%) | 416 (66.8%) | 421 (67.6%) |
| *n (relative %) response^b^* | N/A | 292 (46.9%) | 348 (55.9%) | 364 (58.4%) |
| *n (relative %) remission^c^* | N/A | 199 (31.9%) | 252 (40.4%) | 268 (43.0%) |

^a^MCID (minimal clinically important difference) is defined as an improvement of 4+ points for PHQ/GAD scores, and 5+ points for PCL scores.

^b^Response is defined as 50% or greater improvement in symptoms for PHQ/GAD, or 10+ point improvement for PCL.

^c^Remission was defined as a final symptom score below 5 (for PHQ/GAD); for PCL, Remission” was defined as a 10+ pt improvement combined with a final score below 33.

**^d^PHQ-9 - Patient Health Questionnaire - 9-item**

**^e^GAD-7 - Generalized Anxiety Disorder questionnaire - 7-item**

**^f^PCL-5 - PTSD Checklist for DSM-5.**
